# The Meaning and Purpose Scales (MAPS): development and multi-study validation of short measures of meaningfulness, crisis of meaning, and sources of purpose

**DOI:** 10.1186/s40359-023-01319-8

**Published:** 2023-10-03

**Authors:** Tatjana Schnell, Lars Johan Danbolt

**Affiliations:** 1 MF Norwegian School of Theology, Religion and Society, Oslo, Norway; 2 Institute of Psychology, Existential Psychology Lab, University of Innsbruck, Innsbruck, Austria; 3 Research Centre for Existential Health, Innlandet Hospital Trust, Brumunddal, Norway

**Keywords:** Meaning in life, Crisis of meaning, Sources of meaning, Purpose, Sustainability, Religion, Questionnaire, Financial hardship, German

## Abstract

**Background:**

Meaning in life is multidimensional. It encompasses different qualities of meaning, such as meaningfulness, crisis of meaning, or existential indifference, as well as the sources from which people draw meaning, or purpose. For both research and practice, it is of high value to know not only the extent of meaningfulness, or its absence, but also its sources. How do these relate to meaningfulness and mental health? Are they accessible to people of different sociodemographic and economic backgrounds alike? For therapeutic and counseling practice, knowledge of experiences and sources of meaning is needed to support a clearer self-understanding in patients or clients and to encourage them to make authentic life choices. The Meaning and Purpose Scales (MAPS) presented here enable researchers and practitioners to gain insights into these dimensions of meaning in life, and, with only 23 items, to do so in a short time.

**Methods:**

Using five independent and two follow-up samples with a total *N* of 7,500, this paper examined the MAPS’ internal consistency, test-retest reliability, and convergent, divergent, criterion, factorial, and predictive validity.

**Results:**

Principal axis factoring identified two meaning scales, Meaningfulness and Crisis of Meaning, and five purpose scales, Sustainability, Faith, Security, Community, and Personal Growth. The scales proved consistent, stable over four weeks and two months, and valid in multiple respects. In a representative German population sample, Personal Growth, Sustainability, and Community exhibited large, Faith and Security medium positive relationships with Meaningfulness, whereas Crisis of Meaning showed small to moderate negative correlations. Meaningfulness was positively, and Crisis of Meaning negatively predicted by age, partnership, parenthood, and religious affiliation. Financial hardship correlated positively with Crisis of Meaning and negatively with Meaningfulness, Community, and Personal Growth. Meaningfulness and Crisis of Meaning explained 21%, the sources of purpose 6% of additional variance in general mental distress (PHQ-4), beyond sociodemographics. Except for Faith (unrelated), all sources exhibited moderate negative correlations with the PHQ-4.

**Conclusion:**

As this series of studies demonstrates, the MAPS provide a highly economic and valid assessment of two qualities of meaning, Meaningfulness and Crisis of Meaning, and five sources of purpose: Sustainability, Faith, Security, Community, and Personal Growth.

**Supplementary Information:**

The online version contains supplementary material available at 10.1186/s40359-023-01319-8.

## Background

Correlates and consequences of meaningfulness, or the presence of meaning in life, are well researched. For example, there are marked connections between meaningfulness and mental and physical health [1, 2] and social connectedness [3]. Less is known about sources of meaning, or life’s purposes. This may be because it is relatively difficult to obtain this information. While it is possible to intuitively indicate the degree of (subjective) meaningfulness in one’s life, it is more difficult to state where this meaning derives from, what motivates and guides a person’s actions and decisions, what their ultimate meanings are. According to Leontiev [4, p. 244], “they refer neither to the reality of the surrounding world nor to the reality of individual emotional dynamics, but rather to the reality of links between the individual and the world. Human being in the world has an underlying meaning-based logic of its own.” Embedded in a personal worldview, this “more-or-less coherent whole” of integrated knowledge, experiences, and intuitions constitutes sets of beliefs that guide decision making and action in all spheres of activity [5, p. 3f]. As these beliefs and commitments are generally held sub- or pre-consciously, they ought not to be approached in the same way as declarative knowledge that can be queried directly.

Some studies on sources of meaning relied on plain, direct questions about what gives meaning to life [6] or what constitutes a typically meaningful life [7]. Others used lists of life domains and examined the extent to which they served as sources of meaning [8, 9]. Such approaches appear to evoke rather superficial assumptions. A typical outcome here is that social relationships - and *family* in particular - are named as the most important source of meaning. Phenomenological methods such as laddering questions show that this attribution does not yet reveal much about a person’s experience of meaning, since the term *family* is associated with a wide range of attributions of meaning. The following example from one of our studies will illustrate this. When asked: “What kind of celebrations or ceremonies are of particular importance to you, if any?” a young man answered: “family celebrations”. Asked why this was the case, what they meant to him, he replied: “I enjoy being with my family. They are very funny. Together, we can laugh a lot.” When asked again what this meant to him, he concluded with the following sources of meaning: “relaxation; to let my hair down; to compete a bit with the others, be challenged” [10]. None of these can be inferred from the mere mention of “family celebrations”.

The development of the Sources of Meaning and Meaning in Life Questionnaire, SoMe [11–13] took these “deeper” layers of meaning into account. The questionnaire is based on an extensive qualitative research program, which sought to identify meaning in action by use of interviews, photo studies, observation, and focus groups [14]. This way, sources of purpose emerged by which people - more or less consciously - oriented themselves. Altogether, 26 such sources of purpose were identified, validated through triangulation, and subsequently operationalized as questionnaire scales. These were revised, improved, and extensively validated across different versions [12–14].

The SoMe has been translated into more than 20 languages and is being used in numerous contexts and cultures, but its length is a major obstacle to its wider use. While complex and nuanced procedures are helpful for individual-level decision making, their length is too great an obstacle for screening purposes, for large samples with the primary goal of examining general relationships and differences, or for transdisciplinary research that aims to address lifeworld problems from multiple perspectives [15].

For this reason, an economic adaptation of the SoMe was developed, which is presented here. The Meaning and Purpose Scales (MAPS) reproduce the multidimensional structure of the SoMe, by covering independent measures of meaningfulness, crisis of meaning, and five higher-level dimensions of sources of purpose. The following describes the concepts, the development process, and the studies that tested the scales’ internal consistency, convergent and divergent validity, criterion validity, test-retest reliability, factorial, and predictive validity.

## Conceptualization of the MAPS

### Meaningfulness

Meaningfulness is a basic sense that life is worth living. Current psychological research suggests that it is represented by the following facets: significance/mattering, coherence/comprehension, direction/orientation, and belonging. While some scholars exclude belonging from the conceptualization of meaningfulness [16, 17], others see it at the core of the experience of meaningfulness [18–20], together with significance, coherence, and direction [11, 21]. Belonging, in this context, should not be equated with *social* belonging. The *existential* concept of belonging refers to the sense of having a place in this world, or “dwelling,” as Heidegger [22] called it. “Where one dwells is where one is *at home*, where one *has a place*… [By dwelling, our being] is located within a set of sense-making practices and structures with which it is familiar [23]. In spite of its acknowledgement in European philosophy, the existential significance of belonging is especially emphasized in non-Western cultures, such as in the traditional African concept of “Ubuntu,“ which can be translated as “I am because you are.“ Thus, meaning in life in African contexts is understood as a contextual concept based largely on belongingness [18, 24, 25]. Increasingly, the existential significance of a sense of belonging is also seen cross-culturally, defined as, for example, the background to existence, and experience [26]. As such, it can be understood as a counterpart to the feeling of existential isolation, described by Yalom [27] as one of the four existential givens. Belonging in the existential sense is certainly facilitated by social inclusion, but it can also be enabled in other ways, e.g. through responsibility and caring [28, 29], environmental or political engagement [30], or the feeling body [31].

When it comes to measuring meaningfulness, a valid measure should cover the construct in its breadth. Such domain coverage is crucial for validity, even if it tends to diminish reliability somewhat [32]. The MAPS – just like the SoMe [11, 12], but in contrast to the MLQ [33] – aim to capture the construct of meaningfulness broadly and without redundant items. Hence, the MAPS Meaningfulness scale covers the facets significance, coherence, direction, and belonging, and additionally includes, as an anchor, an explicit statement about the meaningfulness of one’s life.

### Crisis of meaning

A crisis of meaning is defined as a judgement on one’s life as frustratingly empty, pointless, and lacking meaning [11]. The Crisis of Meaning scale explicitly addresses the experience of a lack of meaning, which is clearly distinguishable from the mere absence of meaningfulness: On the one hand, confirmatory factor analyses show that crisis of meaning is not the negative pole of meaningfulness, but that the two constructs are indeed independent [11, 34]; on the other hand, both establish different correlation patterns with a wide range of variables, such as sources of meaning, personality, satisfaction with life, self-efficacy, resilience, trust, religiosity, spirituality, death anxiety, conspiracy mentality, anxiety, depression, general mental distress, PTSD symptoms, psychological burden, or COVID-19 stress [35–43]. The fact that crisis of meaning and meaningfulness can vary independently is further reflected in the phenomenon of existential indifference, characterized by low meaningfulness and low crisis of meaning [44]. While most people who report high meaningfulness also experience low degrees of crisis of meaning, and those who find themselves in a crisis of meaning typically see little meaning in their lives, there is also a substantial number of people (e.g., 18–23% in Germany, cf. [2] and below) who neither experience meaningfulness nor a crisis of meaning. They differ in many respects from the meaningful and crisis of meaning types, e.g., in terms of commitment, competence, self-efficacy, self-esteem, happiness, life satisfaction, hope, or taking responsibility for their lives [2, 45].

### Sources of purpose

Sources of purpose, also termed sources of meaning, “represent a variety of orientations that give meaning to life when being actively pursued. They thus give form to meaning; they are meaning in action” [2, p. 8]. While the extent of personal meaningfulness can be assessed independently of specific sources of meaning, these are needed in order to constitute this very meaning. Meaning seeks to express itself in meaningful action, and meaningful action contributes to a sense of meaningfulness [46].﻿ The orientation of meaningful action is usually not one-dimensional, as several studies on sources of meaning have established [8, 11, 47]. People pursue different goals concurrently, take on diverse roles in life, are involved in different causes. In the course of developing the SoMe, 26 such sources of meaning were identified. Factor-analytically, they can be reduced to four or five dimensions, as multiple studies have replicated [11, 13, 34, 37, 48, 49]. Data repeatedly uncovered four primary dimensions, selftranscendence, selfactualisation, order, and well-being and relatedness. For further explanation of variance, it proved useful and fitting to subdivide selftranscendence into the two subfactors vertical and horizontal selftranscendence. The five superordinate dimensions reflect the breadth of the 26 sources of meaning and can thus act as a reference for the new purpose scales to be developed.

## Studies overview

The following sections present six studies conducted to develop and validate short measures of meaningfulness, crisis of meaning, and sources of purpose. Studies 1 and 2 describe the generation and selection of items, separately for the meaning (Study 1) and purpose scales (Study 2). The next section presents different types of construct validation. Content validity is an indication of whether an instrument actually measures what it claims to measure. In this regard, we were firstly informed by two focus group interviews based on the principles of cognitive interviewing. In addition, content validity was judged by whether the newly developed MAPS measured an appropriate breadth of sources of purpose. Since the (comparable) dimensions of the SoMe were supported by an extensive content validation process, agreement between MAPS and SoMe in this regard suggests content validity. Study 3 examined the convergent and divergent validity of the MAPS based on correlations with the various scales and dimensions of the SoMe. Due to their brevity, the MAPS scales are necessarily narrower than the SoMe dimensions. The specific orientation of the MAPS Sustainability scale was not represented as such in the SoMe. In order to validate the interpretation of this scale as “sustainability”, Study 4 tested it against a criterion measure of pro-environmental behavior. Study 5 employed the new scales in a national sample, representative of the German population 18 + in terms of age, gender, educational background, and residence in the former East or West German states. It offers reliable information on associations between the dimensions of meaning and sociodemographic and economic characteristics, and provides group-specific reference scores (see also appendix). Study 5 further examined the factorial validity of the MAPS using confirmatory factor analyses. Study 6 assessed the short- and medium-term stability of the MAPS by calculating test-retest reliabilities over 4 weeks and 2 months. Finally, we tested the power of the MAPS to predict general mental distress (PHQ-4), beyond sociodemographic and economic variables. Power analyses were calculated using G*Power [50]; confirmatory factor analyses were performed using IBM SPSS AMOS 26; all other analyses were conducted using IBM SPSS Statistics 26.

## Item generation and selection

We used a deductive approach to generate items and a psychometric approach for item selection. The newly developed items were discussed by a larger group of experts and revised for clarity, comprehensibility, and relevance. In the next step, items were selected on the basis of empirical criteria in two separate studies.

### Study 1: Item generation and selection for the Meaningfulness and Crisis of Meaning scales

Study 1 aimed to generate, select, and test items for the MAPS Meaningfulness and Crisis of Meaning scales. The four constructs of significance, coherence, direction, and belonging provided the starting point for the Meaningfulness scale. We intended to select one item of each of the four facets of meaning and one item that explicitly addresses meaningfulness, to ensure a broad but reliable and unidimensional measurement of meaningfulness. A review of the available literature served as a basis for item generation. The present article’s authors devised three items for each of the four facets and the explicit reference to meaningfulness (= 15 items). To create the short Crisis of Meaning scale, three items were phrased in close alignment with the corresponding SoMe scale. They covered aspects of perceived meaninglessness, emptiness, and suffering from a lack of meaning. When drafting the items, we took care to use short and simple sentences on the one hand, but on the other hand to present the core of the respective constructs with the greatest possible specificity and density. To test whether this had been successful, we invited three senior psychological scientists and four graduate students with advanced knowledge of the research subject to evaluate the items’ clarity and comprehensibility, and one of the scientists and two of the students to evaluate if the items covered the desired content, via discussion and written feedback. Based on the responses, several items were reworded. Following this theoretically guided deductive process of item generation, psychometric analyses were used to select the items for the first version of the MAPS.

#### Method

Following the rule of thumb that there should be 10–15 cases per variable when conducting factor analysis [51], we aimed for a sample size of N = 225 (max. 15 variables per analysis). A convenience sample of N = 227 participants completed the 15 meaningfulness and the three crisis of meaning items. Fourteen respondents were excluded due to not stating that they responded honestly to all items. Of the remaining N = 213, 56% were female, 42% male, and 1% diverse; their mean age was 35 years (SD = 17). A six-point Likert-type response format was used (0 = totally disagree to 5 = totally agree). To identify items for the Meaningfulness scale, we used principal axis factoring and extracted a single factor. We then selected the highest loading item per facet. When similar loadings occurred, the item with the highest standard deviation and the lowest skewness was chosen.

#### Results and discussion

Table 1 displays the EFA results for the five selected meaningfulness items after renewed principal axis factoring. The scale is clearly unidimensional (eigenvalues 3.23, 0.67, 0.54).

**Table 1:** Factor loadings for the five items of the Meaningfulness scale

Item	Facet	Meaningfulness factor
1. My life is meaningful.	Meaning	0.87
2. I have found my way.	Direction	0.81
3. My life makes sense to me.	Coherence	0.81
4. I feel connected to this world.	Belonging	0.66
5. My existence enriches the life of others.	Significance	0.59

*Note*. These (and all other) items are ad-hoc translations of the original German items. Principal axis factoring. *N* = 213

Table 2 shows the EFA results for the three crisis of meaning items. They exhibited strong factor loadings and clear one-dimensionality (eigenvalues 2.63, 0.21, 0.15).

**Table 2:** Factor loadings for the three items of the Crisis of Meaning scale

Item	Crisis of meaning factor
1. I am missing meaning in my life.	0.93
2. I suffer because I can’t see any meaning in my life.	0.91
3. My life seems empty to me.	0.87

*Note.* Principal axis factoring. *N* = 213

### Study 2: Item generation and selection for the sources of purpose scales

**Fig. 1:**
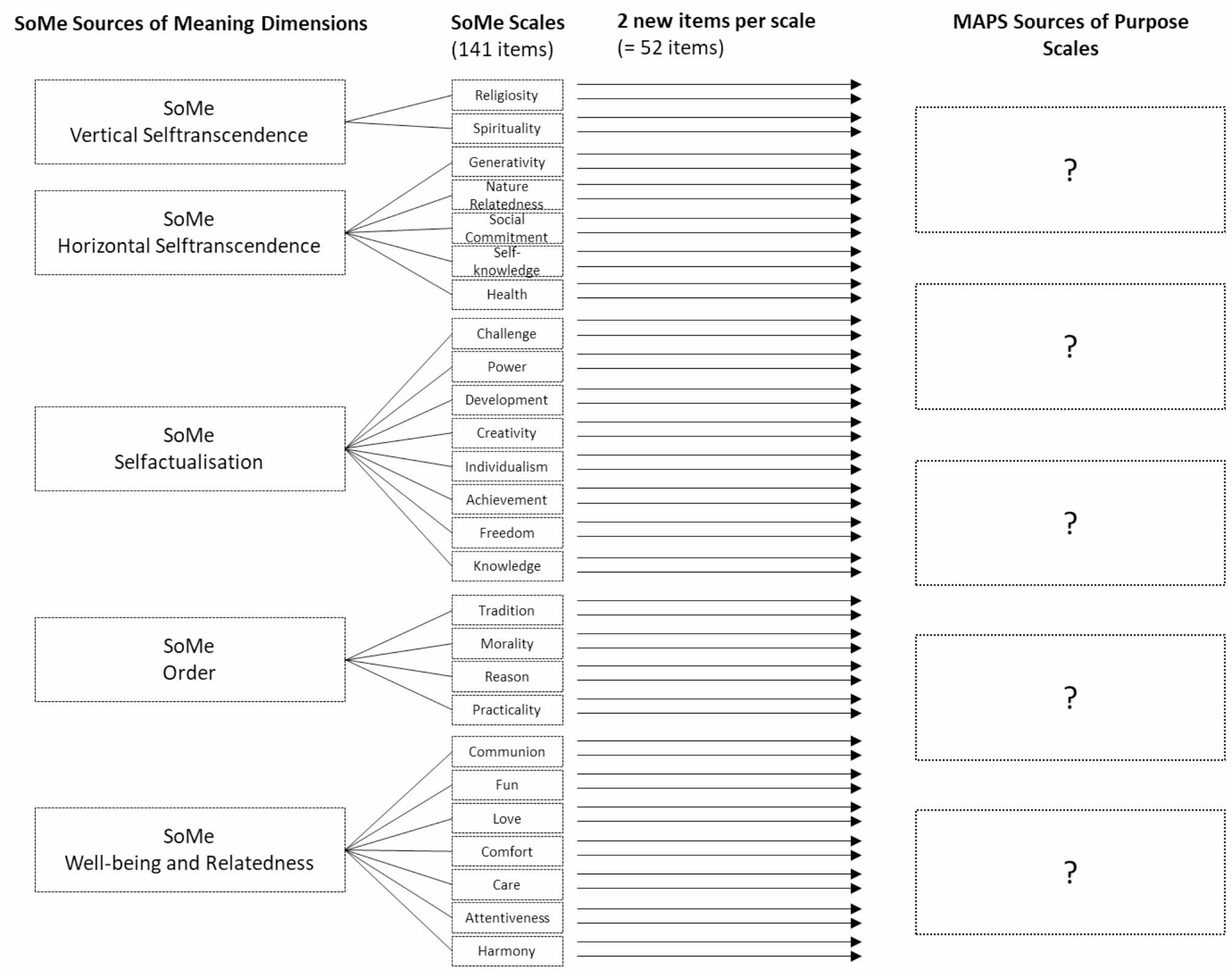
Generating sources of purpose items with reference to the SoMe sources of meaning scales

The SoMe scales were used as a starting point for the sources of purpose scales. To ensure that the targeted sources of purpose scales drew on the full range of sources covered by the SoMe, each of the SoMe scales was represented by two new items. These were phrased with specific reference to the content of those items that had, in earlier factor analyses, shown the highest loadings per scale. In this way, 52 new items were developed, following the same criteria as described in Study 1. Feedback on clarity, comprehensibility, and relevance was sought from three journalists. Based on discussions and written comments, the wording of several items was refined. In a next step, the 52 items were subjected to principal axis factoring. Figure 1 illustrates the process.

#### Method

Data were collected in collaboration with a public service broadcaster. A total of N = 13,686 German participants over 18 years responded to the newly developed 52 sources of purpose items. A six-point Likert-type response format was used (0 = totally disagree to 5 = totally agree). After excluding participants with incomplete responses or age specification > 99, an effective N = 13,660 ensued. According to [50], sample sizes of 1,000 or more are deemed excellent for factor analysis. We therefore decided to draw a random subsample of N = 3,000. 57% were male, and the average age was 34 years (SD = 13). We conducted principal axis factoring and, based on the pattern matrix, identified three items per factor that had high loadings and no double loadings >|0.3|, high standard deviations, and that covered the construct as broadly as possible while still maintaining sufficient internal consistency. In a next step, the selected items were again subjected to principal axis factoring, but – to ensure that the factor structure applies to both men and women - separately for both genders. To this end, two additional random samples of N = 1,500 respondents each were drawn from the total sample. The women’s mean age was 33 (SD = 12), and the men’s mean age was 35 (SD = 13).

#### Results and discussion

In the joint factor analysis, the items loaded clearly on five factors. For each factor, three items were identified that fulfilled the above-described criteria: Three items represent the dimension ‘Sustainability’ - a sense of connectedness with all forms of life and concern for a future worth living; further three items describe the religious/spiritual dimension ‘Faith’ - a sense of connectedness with transcendence and concern for a spiritual life; the next three items cover a dimension we called ‘Security’ - a sense of connectedness with shared norms and concern for a secure life; another three items stand for the social dimension ‘Community’ - a sense of connectedness with a familiar group and concern for each other, and the last three items belong to the dimension we labeled as ‘Personal Growth’ - a sense of connectedness with one’s self and concern for continuous learning. The following factor analyses, conducted for the female and male subsamples, replicated the factor structure. Table 3 shows the factor loadings for both samples. Eigenvalues for the female sample were 3.22, 1.99, 1.82, 1.50, 1.30, 0.73. For the male sample, they were 3.75, 1.97, 1.70, 1.40, 1.20, 0.74.

**Table 3:** PAF factor loadings for the five sources of purpose scales, separately for women and men

Item	Scale	1	2	3	4	5
a) Female sample, *N* = 1,500						
Sus_1	Sustainability	**0.86**				
Sus_2		**0.62**	0.11			
Sus_3		**0.60**			0.15	
Fai_1	Faith		**0.89**			
Fai_2		0.11	**0.75**			
Fai_3			**0.62**			
Sec_1	Security			**0.70**		
Sec_2				**0.65**		
Sec_3				**0.63**		
Com_1	Community				**0.76**	
Com_2					**0.64**	
Com_3		0.15			**0.51**	
Per_1	Personal Growth					**0.72**
Per_2						**0.69**
Per_3						**0.58**
b) Male sample, *N* = 1,500						
Per_1	Personal Growth	**0.70**			0.10	
Per_2		**0.67**				
Per_3		**0.65**				
Fai_1	Faith		**0.91**			
Fai_2			**0.80**		0.11	
Fai_3			**0.72**			0.10
Sec_1	Security	− 0.15		**0.66**		
Sec_3				**0.60**		
Sec_2		0.22		**0.57**		
Sus_1	Sustainability				**0.82**	− 0.11
Sus_2					**0.59**	
Sus_3					**0.54**	0.21
Com_2	Community					**0.70**
Com_1		0.13			− 0.10	**0.60**
Com_3					0.25	**0.55**

*Note.* Principal axis factoring, oblimin rotation

## Construct validity

“Construct validity is the degree to which an assessment instrument measures the targeted construct… [It] subsumes all categories of validity” [52, p. 239]. We address the following types of validity: content validity, convergent and divergent validity, criterion, factorial, and predictive validity.

### Content validity

As part of content validation, the empirically selected MAPS items were evaluated through cognitive interviewing principles in two focus groups. To engage the perspectives of a wide variety of users, participants were drawn from non-academic contexts and represented different educational and age groups. They had the task of paraphrasing the items, explaining individual terms, and sharing their interpretation of the items. As a result, five of the sources of purpose items (but none of the meaningfulness and crisis of meaning items) were reworded for the sake of semantic precision. (The final German and English versions of the MAPS are shown in Tables A1 and A2 in the appendix.)

The content validity of the MAPS is further supported by the fact that the instrument reflects the content and higher-order structure of the SoMe. Since the items and scales of the SoMe were developed during a four-month test design workshop and rigorously checked for content validity [13], these steps were not repeated for the MAPS. The newly developed MAPS items replicate the five-factor structure of the SoMe (see Fig. 1), and the MAPS scales overlap with the SoMe dimensions and scales to the expected degree, as will be shown in the following.

### Study 3: Convergent and divergent validity

As with the SoMe, a validated albeit much longer measure of the targeted constructs is available, the new instrument was first validated against the existing one.

#### Method

Power analysis [G*Power, 50] suggested a sample size of N = 84 to detect medium effects with 80% probability (two-sided). Because we planned to invite study participants to repeat the test in order to calculate test-retest reliability, we oversampled due to the expected drop-out and aimed for a sample size of at least N = 100. N = 128 students completed both the revised version of the MAPS (including the five reworded items) and the SoMe. Three participants were excluded due to missing data, and eight participants due to not stating that they answered all questions honestly. Of the remaining N = 117, 66% were female, 32% male, and 2% diverse. The mean age was 26 years (SD = 10). The Sources of Meaning and Meaning in Life Questionnaire [12, 13] measures meaningfulness (5 items), crisis of meaning (5 items), and 26 sources of meaning (141 items). All items are rated on a six-point Likert scale (0 – totally disagree; 5 – totally agree). The questionnaire has proven to be reliable and valid in multiple contexts and cultures [12, 34, 37, 39, 42, 53–55]. We expected very high correlations (r > .70) between the MAPS and the SoMe Meaningfulness and Crisis of Meaning scales and high correlations (r > .50) between the MAPS sources of purpose scales and the respective SoMe dimensions. Because the latter are significantly broader, less overlap with the new, reduced MAPS scales was expected.

#### Results and discussion

As hypothesized, the Meaningfulness and Crisis of Meaning scales of the MAPS overlapped with the respective SoMe scales to a very high degree (see Table 4). The five short MAPS sources of purpose scales also correlated strongly with the much longer and more heterogeneous SoMe dimensions, confirming the intended alignment with the structure and content of the SoMe and the scales’ convergent validity. Of the five SoMe dimensions, vertical selftranscendence is the narrowest, as it only represents two sources of meaning, religiosity and spirituality. This reflects in the very high correlation with the MAPS Faith scale, which measures a general connectedness with transcendence and concern for a spiritual life. Correlations between the MAPS sources of purpose scales and other SoMe dimensions are lower throughout, thus confirming the divergent validity of the sources of purpose scales.

**Table 4:** Correlations between MAPS and SoMe constructs

MAPS → SoMe ↓	Meaningfulness	Crisis of Meaning	Faith	Sustainability	Personal Growth	Security	Community
Meaningfulness	**0.80** ^***^	− 0.77^***^	0.48^***^	0.43^***^	0.51^***^	0.03	0.37^***^
Crisis of meaning	− 0.81^***^	**0.93** ^***^	− 0.19^*^	− 0.36^***^	− 0.48^***^	0.11	− 0.28^**^
Vertical selftranscendence	0.34^***^	− 0.24^*^	**0.93** ^***^	0.23^*^	0.20^*^	0.12	0.12
Horizontal selftranscendence	0.49^***^	− 0.37^***^	0.32^***^	**0.69** ^***^	0.49^***^	0.05	0.25^**^
Selfactualisation	0.26^**^	− 0.22^*^	0.10	0.37^***^	**0.52** ^***^	− 0.14	0.10
Order	0.10	0.13	0.41^***^	0.01	0.05	**0.58** ^***^	0.13
Well-being and relatedness	0.44^***^	− 0.27^**^	0.21^*^	0.23^*^	0.23^*^	− 0.02	**0.69** ^***^

*Note*. Bold = expected overlaps. *N* = 117

^*^p < .05 (two-sided). ^**^p < .01 (two-sided). ^***^p < .001 (two-sided)

### Study 4: Criterion validity

The Sustainability scale introduces a – relative to the SoMe dimensions – new perspective to the MAPS by combining items that tap the SoMe scales nature relatedness and generativity. They address “living in harmony with nature,” “experiencing connectedness with all living beings,” and “basing actions on leaving a world worth living in for future generations.” With a high degree of face validity, these items address sustainability as defined, e.g., by the United States Environmental Protection Agency [56]: “To pursue sustainability is to create and maintain the conditions under which humans and nature can exist in productive harmony to support present and future generations.” To further test the (criterion) validity of this ‘new’ construct, we examined its capacity to predict self-reported pro-environmental behavior. A positive correlation of medium to large size was expected.

#### Method

Power analysis [50] suggested a sample size of N = 37 to detect a medium to large effect (r = .40) with 80% probability (one-sided). A sample of *N* = 61 students from a wide range of disciplines completed the MAPS and provided information on the frequency with which they practiced six aspects of pro-environmental behavior (0 = never to 5 = always):

- Buying organic food.
- Limiting consumption of meat.
- Saving water at home for environmental reasons.
- Taking care to close windows and doors to save energy.
- Shutting down/switching off electronic devices after use.
- Using lower doses of detergent than recommended by the manufacturer.

Three of the participants were excluded due to not stating that they answered all questions honestly. Of the remaining *N* = 58, 60% were female, 38% male, 2% diverse. The mean age was 22 years (*SD* = 6).

#### Results and discussion

The items intercorrelated highly enough to permit aggregation (Cronbach’s alpha = 0.70, McDonald’s omega = 0.71). The resulting measure of pro-environmental behavior and the MAPS Sustainability scale correlated at *r* = .44 (*p* = .001), whereas correlations with Faith, Security, Community, and Personal Growth were non-significant (*r*s = .-0.10, 0.06, − 0.14, 0.06). The medium-to-high association between pro-environmental behavior and Sustainability underlines the scale’s concurrent criterion validity.

## Study 5: Descriptive statistics and sociodemographics

A national population survey was conducted to provide comprehensive descriptive statistics and balanced reference scores. Additionally, the breadth of the sample allows for reliable statements about associations with sociodemographic and economic characteristics.

### Method

To recruit a balanced sample, we collaborated with a professional survey company, Consumerfieldwork GmbH, with over 39,000 panelists across Germany at the time of recruitment (October 2021). A sample size of N = 1,000 has proven sufficient to adequately represent the German population (approx. 70 million adults) [57]. Our initial sample included N = 1,000 participants. It was representative of the German population 18 + in terms of age, gender, educational background (medium level qualification, higher education entrance qualification, university degree), and residence in the former East or West German states. Again, participation was entirely voluntary. Participants were paid a small remuneration for questionnaire completion. Before analysis, we excluded n = 21 respondents with excessively short response times (relative speed index > 2; [58]), n = 1 duplicate case, and n = 4 participants with repetitive responses for more than ten items.

Hence, the final sample included N = 974 subjects. Of these, 51% self-identified as female, 49% as male. The participants’ age ranged from 18 to 89, with a mean of 50 years (SD = 16). 63% were partnered (41% married, 22% single, 21% in a committed relationship, 10% separated or divorced, 5% widowed, 1% in a registered partnership), 56% had one or more children. Highest educational levels were distributed as follows: secondary and intermediate secondary school certificates: 70%; higher education entrance qualification: 14%; university degree: 16%. 15% of the respondents came from former East German states, 80% from former West German states, 5% from Berlin. 58% identified as belonging to one of the five world religions (51% Christian, 5% Buddhist, 2% Islam, 0.3% Judaism). 57% reported having “some”, “considerable”, or “great” difficulty in making ends meet with their total monthly income (Deleeck question; cf. [59]).

We report mean and standard deviation scores, number of items, response range, skewness, range of corrected item-total correlations, and scale reliabilities, as well as scale intercorrelations and correlations with the sociodemographic variables. For estimating reliability, we used both Cronbach’s alpha and McDonald’s omega (with 95% CIs), as the latter is advocated as a related but better alternative [60], especially for short scales [61].

### Results and discussion

Table 5 displays the MAPS’ descriptive statistics. Skewness values <|2| indicate near-normal distribution [62]. As Crisis of Meaning nevertheless showed a strong positive skew, the median is reported in addition to the mean. All corrected item-total correlations are > 0.52; they thus markedly exceed the required minimum value of *r* = .30. Reliability scores range from 0.74 (Cronbach’s alpha) or 0.75 (McDonald’s omega) to 0.96 and can thus be considered good to excellent, especially given the small number of items.

Interpreting the absolute values, the population exhibited a relatively high sense of meaning, and low levels in crisis of meaning. Combining both scales [44], the following distribution of types of meaning emerged: 68% meaningful, 18% existentially indifferent, 14% in a crisis of meaning (including 3% who also reported positive [> 3] meaningfulness). Compared to Community, Personal Growth, Security, and Sustainability, commitment to Faith was low. Table A3 in the appendix shows age-specific reference scores for the MAPS and three types of meaning. Table A4 in the appendix delineates means and standard deviations for responses to the individual MAPS questions.

**Table 5:** Scale means and standard deviations, number of items, response range, skewness, range of corrected item-total correlations, and internal consistencies (McDonald’s Omega and Cronbach’s alpha) with 95% CIs

	Mean [Med]	*SD*	No. of items	Skewness	Range corrected item-total correlations	McDonald’s omega with 95% *CI* [*LL, UL*]^a^	Cronbach’s alpha with 95% *CI* [*LL, UL*]^a^
Meaningfulness^b^	3.37	0.91	5	− 0.60	0.60 − 0.83	0.89 [0.88, 0.91]	0.89 [0.88, 0.90]
Crisis of Meaning^b^	1.19 [0.67]	1.30	3	1.02	0.76 − 0.81	0.89 [0.87, 0.91]	0.89 [0.87, 0.91]
Sustainability^b^	3.08	1.11	3	− 0.39	0.59 − 0.68	0.79 [0.76, 0.82]	0.79 [0.76, 0.81]
Faith^b^	1.47	1.68	3	0.83	0.89 − 0.92	0.96 [0.95, 0.96]	0.96 [0.95, 0.96]
Security^b^	3.30	0.90	3	− 0.50	0.54 − 0.59	0.75 [0.71, 0.77]	0.74 [0.71, 0.78]
Community^b^	3.44	1.02	3	− 0.67	0.53 − 0.66	0.77 [0.75, 0.80]	0.76 [0.73, 0.79]
Personal Growth^b^	3.43	1.00	3	− 0.54	0.58 − 0.69	0.81 [0.78, 0.83]	0.80 [0.77, 0.83]

*Note*. *N* = 974

*LL* = lower level. *UL* = upper level. ^a^ Confidence intervals for McDonald’s omega (HA) and Cronbach’s alpha obtained by using OMEGA [60]. ^b^ Response range 0–5

As shown in Table 6, all correlations between the meaning and purpose scales were significant, with medium to large effects for relationships between Meaningfulness and the five sources of purpose scales and small to medium effects for correlations between Crisis of Meaning and the five sources of purpose scales. The (negative) associations between Crisis of Meaning and sources of meaning are known to be smaller than the (positive) associations between Meaningfulness and sources of meaning [2, 37, 42], since the absence of a crisis of meaning does not necessarily imply a commitment to a source of purpose, and even when there is a crisis of meaning, isolated commitments may still be present. Intercorrelations between the sources of purpose scales ranged from *r* = .08 to *r* = .54.

Regarding sociodemographics and the two meaning scales, higher age, being partnered, having children, and being religious were associated with higher meaningfulness and lower crisis of meaning. This is in line with the literature cf. [2]. Further differentiation with respect to marital status replicates the finding that singles report the lowest levels of meaningfulness and the highest levels of crisis of meaning (estimated marginal means, controlling for age: *M* = 3.09, *SE* = 0.06 and 1.59, *SE* = 0.09, resp.). As also previously found, married persons reported higher meaningfulness than cohabitants (*M* = 3.55, *SE* = 0.04 vs. 3.32, *SE* = 0.06). Gender, education, and residence in the former East vs. West German states were unrelated. Financial hardship was negatively related with meaningfulness, and positively related with crisis of meaning (moderate effects).

Age was positively related with commitments to Sustainability, Faith, and Security (small to moderate effects). Women were slightly more committed to both Faith and Community than men. This replicates the well-known “religious/spiritual gender gap” [63, 64] and the finding that women are more communal (but not less agentic – see Personal Growth) than men [65]. People who lived with (any kind of) partner also exhibited a slightly stronger commitment to Community – as did people with children. The latter also reported slightly higher commitments to Sustainability, Faith, and Personal Growth. Higher education was associated with higher commitment to Personal Growth and Community, and lower commitment to Security, although the last two correlations were minimal. Apart from one moderate effect (Faith) that mirrored the traditionally higher secularity in the East, no differences between residents in the former East vs. West German states were found. Religious belonging was strongly positively related to Faith, but also – with small to moderate effects – to Community, Personal Growth, Sustainability, and Security. Finally, the more financial hardship people reported, the less they were committed to Community and Personal Growth (small effects).

**Table 6:** Scale intercorrelations and correlations with sociodemographic and economic variables

	Meaningfulness	Crisis of Meaning	Sustainability	Faith	Security	Community	Personal Growth
Meaningfulness	**--**	− 0.67^***^	0.60^***^	0.29^***^	0.32^***^	0.60^***^	0.69^***^
Crisis of Meaning		**--**	− 0.29^***^	− 0.08^*^	− 0.13^***^	− 0.27^***^	− 0.39^***^
Sustainability			**--**	0.37^***^	0.24^***^	0.45^***^	0.54^***^
Faith				**--**	0.08^**^	0.25^***^	0.30^***^
Security					**--**	0.25^***^	0.20^***^
Community						**--**	0.49^***^
Age	0.21^***^	− 0.26^***^	0.28^***^	0.11^**^	0.11^**^	0.06	− 0.00
Gender^a^	0.00	− 0.02	− 0.02	− 0.15^***^	− 0.07^*^	− 0.12^***^	− 0.06
Partnered^b^	0.16^***^	− 0.17^***^	0.04	0.01	0.02	0.15^***^	0.06
Children^c^	0.20^***^	− 0.20^***^	0.18^***^	0.13^***^	0.05	0.21^***^	0.09^**^
Education^d^	0.02	0.02	0.00	− 0.04	− 0.07^*^	0.07^*^	0.14^***^
East vs. West^e^	− 0.03	0.03	− 0.01	0.15^***^	− 0.00	0.01	0.01
Religion^f^	0.19^***^	− 0.08^*^	0.16^***^	0.48^***^	0.08^*^	0.22^***^	0.18^***^
Financial hardship^g^	− 0.23^***^	0.28^***^	− 0.02	0.04	− 0.06	− 0.11^**^	− 0.10^**^

*Note*. *N* = 974

^a^ 1 = female, 2 = male. ^b^ 1 = not partnered (single, divorced, widowed), 2 = partnered (marriage, legalized partnership or cohabitation). ^c^ 0 = no children, 1 = 1 or more children. ^d^ Secondary, Secondary advanced, Higher education entrance qualification, Bachelor degree, Master degree, Doctorate. ^e^ 1 = former East, 2 = former West German states (including Berlin). ^f^ 1 = secular (humanist, atheist, agnostic, no affiliation). 2 = religious (Christianity, Buddhism, Islam, Judaism). ^g^ Difficulty in making ends meet with total monthly income (0–5). ^*^p < .05 (two-sided). ^**^p < .01 (two-sided). ^***^p < .001 (two-sided)

## Factorial validity

The sample from Study 5 was used to confirm the factorial validity of the MAPS across different demographic groups.

### Method

CFA factor models were estimated (ML, IBM SPSS AMOS 26) for the total sample (*N* = 974), for women (*n* = 499) and men (*n* = 475), and for two age groups (18–50, *n* = 467; 51–89, *n* = 507). The sample sizes can be considered sufficient for conducting CFAs [66]. For each sample, meaning and purpose models were estimated separately, as they represent different levels. For the Meaningfulness and Crisis of Meaning scales, both one- and two-factor models were estimated to examine the hypothesized better fit of the two-factor solution. Chi-square difference tests between both models followed.

### Results and discussion

Table 7 displays the goodness-of-fit indices chi-square, comparative fit index (CFI), standardized root mean-square residual (SRMR), and root mean-square error of approximation (RMSEA). When two models are being compared, Akaike’s information criterion (AIC) and Schwarz’s Bayesian information criterion (BIC) are also reported. All error terms are uncorrelated. In each sample, the two-factor model for Meaningfulness and Crisis of Meaning exhibited a better fit than the competing one-factor model, as evidenced by the lower AIC and BIC coefficients and the following χ^2^ difference tests [67]: For the total sample, χ^2^ (1) = 492.34, *p* < .001, for women, χ^2^ (1) = 258.39, *p* < .001, for men, χ^2^ (1) = 232.57, *p* < .001, for the younger age-group χ^2^ (1) = 197.27, *p* < .001, and for the older age group, χ^2^ (1) = 288.88, *p* < .001. For all hypothesized models, the CFI values were 0.95 or higher, thus indicating acceptable (≥ 0.95) to good (≥ 0.97) fit. The same holds for the SRMR values, which ranged from acceptable (0.056 − 0.051) to good (0.046 − 0.031). The RMSEA scores can all be deemed acceptable [67].

**Table 7:** Goodness-of-fit statistics for the meaning and purpose scales in different subsamples

Sample	Model	χ2 (df)	CFI	SRMR	RMSEA [90% CI]	AIC	BIC
Total	Two-factor Meaningfulness, Crisis of Meaning scales	102.79 (19)	**0.98**	**0.030**	**0.067 [0.055, 0.080]**	**136.79**	**219.78**
	One-factor Meaningfulness, Crisis of Meaning scales	595.13 (20)	0.89	0.063	0.172 [0.160, 0.184]	627.13	705.23
	Five-factor sources of purpose scales	345.12 (80)	**0.97**	**0.045**	**0.058 [0.052, 0.065]**		
Women	Two-factor Meaningfulness, Crisis of Meaning scales	61.67 (19)	**0.99**	**0.031**	**0.067 [0.049, 0.086]**	**95.67**	**167.29**
	One-factor Meaningfulness, Crisis of Meaning scales	320.06 (20)	0.90	0.060	0.174 [0.157, 0.191]	352.06	419.46
	Five-factor sources of purpose scales	262.72 (80)	**0.95**	**0.051**	**0.068 [0.059, 0.077]**		
Men	Two-factor Meaningfulness, Crisis of Meaning scales	70.52 (19)	**0.98**	**0.033**	**0.076 [0.057, 0.095]**	**104.52**	**175.29**
	One-factor Meaningfulness, Crisis of Meaning scales	303.09 (20)	0.88	0.068	0.173 [0.156, 0.190]	335.09	401.70
	Five-factor sources of purpose scales	207.77 (80)	**0.96**	**0.046**	**0.058 [0.048, 0.068]**		
Age 18–50	Two-factor Meaningfulness, Crisis of Meaning scales	58.12 (19)	**0.98**	**0.031**	**0.066 [0.047, 0.086]**	**92.12**	**162.61**
	One-factor Meaningfulness, Crisis of Meaning scales	255.39 (20)	0.99	0.058	0.159 [0.142, 1.77]	287.39	353.73
	Five-factor sources of purpose scales	240.12 (80)	**0.95**	**0.056**	**0.066 [0.056, 0.075]**		
Age 51–89	Two-factor Meaningfulness, Crisis of Meaning scales	81.27 (19)	**0.98**	**0.035**	**0.080 [0.063, 0.099]**	**115.27**	**187.15**
	One-factor Meaningfulness, Crisis of Meaning scales	370.15 (20)	0.88	0.074	0.186 [0.170, 0.203]	402.15	469.81
	Five-factor sources of purpose scales	216.95 (80)	**0.97**	**0.046**	**0.058** **[0.049, 0.068]**		

*Note*. Total: *N* = 974. Women: *n* = 499. Men: *n* = 475. Age 18–50: *n* = 467. Age 51–89: *n* = 507. Bold = hypothesized model

## Study 6: test-retest reliability

Test-retest reliability of the MAPS was examined across two different time intervals: four weeks and two months.

### Method

Out of the *N* = 128 students from Study 3, *n* = 38 completed the MAPS again after four weeks. A sample of *N* = 23 is sufficient for detecting large effects (one-sided) with a statistical power of 80% [50]. Of those who participated both times, 29% were male, 68% female, 3% diverse; mean age was 26 years (*SD* = 12). A random subsample of the *N* = 1,000 participants in Study 5 also completed the MAPS a second time. Here, the time interval was two months. Of those *n* = 100 who completed both surveys, 46% were female and 54% were male. Their mean age was 46 years (*SD* = 16).

### Results and discussion

For both time intervals, all scales exhibited excellent (≥ 0.75; [68]) test-retest reliability (see Table 8).

**Table 8:** Test-retest-reliability after four weeks (n = 38) and two months (n = 100)

Variable	Test-retest reliability (4 weeks, *n* = 38)	95% *CI* [*LL, UL*]	Test-retest reliability (2 months, *n* = 100)	95% *CI* [*LL, UL*]
Meaningfulness	0.84	[0.71, 0.91]	0.84	[0.78, 0.89]
Crisis of Meaning	0.92	[0.85, 0.96]	0.80	[0.72, 0.86]
Sustainability	0.85	[0.73, 0.92]	0.89	[0.84, 0.92]
Faith	0.96	[0.93, 0.98]	0.91	[0.87, 0.94]
Security	0.81	[0.66, 0.90]	0.77	[0.68, 0.84]
Community	0.76	[0.58, 0.87]	0.83	[0.76, 0.88]
Personal Growth	0.78	[0.62, 0.88]	0.79	[0.71, 0.86]

*Note*. Confidence interval estimation = Fisher. *LL* = lower level. *UL* = upper level

## Predictive validity: meaning in life predicts general mental distress

Finally, to demonstrate the relevance of the measured constructs for the clinical context, the MAPS were used to predict general mental distress. Numerous studies have shown that meaningfulness is positively associated with mental health, and crisis of meaning negatively (for a summary, see [2]). Both findings shall be replicated here by examining the new scales’ predictive validity concerning general mental distress, while controlling for sociodemographics. Less is known about the relationships between different sources of meaning, or purpose, and mental health. First findings suggest that a commitment to a purpose is not per se related to mental health or ill-health. Most sources of meaning showed small to moderate negative associations with depression and/or anxiety, whereas religion – or vertical selftranscendence – stood out for being repeatedly unassociated with mental distress [11, 37, 69].

### Method

Also this data was taken from Study 5. As a measure of general mental distress, the Patient Health Questionnaire-4 (PHQ-4; [70]) was employed. The brief four-item scale measures core symptoms of depression and anxiety (four-point Likert scale, 0–3). In the present study, Cronbach’s alpha and McDonald’s omega both were 0.88. We conducted two hierarchical linear regressions to predict general mental distress: The first included Meaningfulness and Crisis of Meaning as predictors, the second included the five sources of purpose scales, Sustainability, Faith, Security, Community, and Personal Growth. In both regressions, sociodemographic and economic variables were included in the first (model 1) and meaning variables were added in the second block (model 2). Statistical power analysis [50] suggests that the given *N* = 974 is large enough to detect even small effects.

### Results and discussion

Model 1 (the same in both regressions) predicted general mental distress from sociodemographics and financial hardship, which accounted for a significant amount of variance, *F*(8,965) = 32.31, *p <* .001, *R*^*2*^ = 0.21. The addition of Meaningfulness and Crisis of Meaning in the second model, *F*(10,963) = 91.46, *p* < .001, *R*^*2*^ = 0.49, showed significant improvement from the first model, ∆*F*(2,963) = 258.95, *p* < .001, *∆R*^*2*^ = 0.28. Both Meaningfulness and Crisis of Meaning captured unique variance in the outcome variable, beyond sociodemographics (see Table 9). For every 1-unit increase in perceived Meaningfulness, symptoms of depression and anxiety are expected to decrease by 0.61 units, holding all other predictors constant. For a 1-unit increase in Crisis of Meaning, mental distress is expected to increase 1.01 units, holding all other predictors constant.

Comparable with previous studies, the sources of purpose scales were less predictive of general mental distress. Their inclusion in the second model, *F*(13,960) = 27.37, *p* < .001, *R*^*2*^ = 0.27, resulted in a significant improvement from the first model, ∆*F*(5,960) = 15.56, *p* < .001, but this only led to an additional explanation of 6%. Although all sources of purpose but Faith established small to moderate negative zero-order correlations with general mental distress, only Personal Growth served as a significant predictor in the regression. For every 1-unit increase in Personal Growth, general mental distress is expected to decrease 0.65 units, holding all other predictors constant.

The findings thus suggest that a commitment to Personal Growth predicts better mental health, whereas Sustainability, Security, and Community might be associated with lower general mental distress, but did not show a predictive power thereof.

**Table 9:** Two hierarchical multiple regressions to predict general mental distress

Model	Predictors	*B*	*SE B*	95% CI for *B*		β	*r*_zero order_	*R* ^*2*^ *(ΔR* ^*2*^ *)*
				*LL*	*UL*			
1	Intercept	3.45^***^	0.75	1.98	4.92			
	Age	-0.05^***^	0.01	− 0.06	− 0.04	− 0.27	− 0.28^***^	
	Gender	-0.51^**^	0.18	− 0.85	− 0.16	− 0.09	− 0.13^***^	
	Partnered	-0.19	0.18	− 0.55	0.18	− 0.03	− 0.11^***^	
	Children	-0.12	0.09	− 0.30	0.05	− 0.04	− 0.15^***^	
	Education	0.00	0.05	− 0.09	0.09	0.00	0.02	
	East vs. West	0.36	0.25	− 0.12	0.85	0.04	0.02	
	Religion	-0.35	0.18	− 0.70	0.01	− 0.06	− 0.08*	
	Financial hardship	0.82^***^	0.08	0.68	0.97	0.33	0.34^***^	0.21
2	MEANING SCALES							
	Intercept	4.20^***^	0.73	2.77	5.62			
	Age	-0.02^***^	0.00	− 0.03	− 0.01	− 0.12	− 0.28^***^	
	Gender	-0.59^***^	0.14	− 0.87	− 0.31	− 0.10	− 0.13^***^	
	Partnered	0.16	0.15	− 0.13	0.46	0.03	− 0.11^***^	
	Children	0.03	0.07	− 0.12	0.17	0.01	− 0.15^***^	
	Education	0.02	0.04	− 0.05	0.10	0.02	0.02	
	East vs. West	0.01	0.20	− 0.39	0.40	0.00	0.02	
	Religion	-0.02	0.15	− 0.32	0.27	0.00	− 0.08^*^	
	Financial hardship	0.44^***^	0.06	0.31	0.56	0.17	0.34^***^	
	**Meaningfulness**	-0.61^***^	0.10	− 0.81	− 0.40	− 0.19	− 0.54^***^	
	**Crisis of Meaning**	1.01^***^	0.07	0.86	1.15	0.44	0.64^***^	0.49 (0.28)
2	PURPOSE SCALES							
	Intercept	6.53^***^	0.84	4.88	8.17			
	Age	-0.05^***^	0.01	− 0.06	− 0.04	− 0.26	− 0.28^***^	
	Gender	-0.61^***^	0.17	− 0.94	− 0.27	− 0.10	− 0.13^***^	
	Partnered	-0.12	0.18	− 0.47	0.23	− 0.02	− 0.11^***^	
	Children	-0.06	0.09	− 0.24	0.11	− 0.02	− 0.15^***^	
	Education	0.05	0.05	− 0.04	0.14	0.03	0.02	
	East vs. West	0.26	0.24	− 0.21	0.73	0.03	0.02	
	Religion	-0.18	0.20	− 0.57	0.21	− 0.03	− 0.08^*^	
	Financial hardship	0.76^***^	0.07	0.62	0.91	0.30	0.34^***^	
	**Sustainability**	0.02	0.10	− 0.17	0.22	0.01	− 0.21^***^	
	**Faith**	0.10	0.06	− 0.02	0.22	0.06	− 0.04	
	**Security**	-0.12	0.10	− 0.30	0.07	− 0.04	− 0.13^***^	
	**Community**	-0.18	0.10	− 0.38	0.01	− 0.06	− 0.21^***^	
	**Personal Growth**	-0.65^***^	0.11	− 0.86	− 0.44	− 0.22	− 0.26^***^	0.27 (0.06)

*Note*. *N* = 974. *LL* = Lower level. *UL* = Upper level

* *p* < .05 (two-sided). ** *p* < .005 (two-sided). *** *p* < .001 (two-sided)

## General discussion

The present article describes the process from the rationale and development of the Meaning and Purpose Scales (MAPS), short measures of Meaningfulness, Crisis of Meaning, and sources of purpose, to several studies that tested their reliability and validity. The availability of such an instrument is considered important for several reasons. Research interest in meaning in life has increased greatly in recent years (e.g., number of hits with “meaning in life” in the title in Google Scholar, 1992–2001 = 193, 2002–2011 = 588, 2012–2021 = 2,200). Numerous studies address relationships between meaning in life and various psychological constructs. Most of them deal with the experience of meaning, such as presence of meaning, meaningfulness, search for meaning, crisis of meaning, or existential indifference. Less frequently, sources of meaning, or purpose, are investigated. The instrument presented here will enable this multidimensional construct to be integrated into various research programs in a highly economic but valid way. Alongside a need for short measures in large-sample research seeking to understand more general relationships, short measures are also beneficial for transdisciplinary research. Transdisciplinary research programs are the means of choice when investigating complex lifeworld problems [15, 71]. Considering the experience - or lack - of meaning and related sources of purpose will provide crucial insights in many areas such as health, the workplace, education, ethics, economics, politics, and many others. Last but not least, short measures are instrumental as screening instruments in the psychological field, for example, at the beginning of therapy or counseling.

### Summary of the present studies

The MAPS are designed to measure Meaningfulness - a basic sense that life is worth living, based on the (mostly unconscious) evaluation of one’s life as significant, coherent, directed, and belonging; Crisis of Meaning - a judgement on one’s life as frustratingly empty, pointless, and lacking meaning, and sources of purpose - orientations that give meaning to life when actively pursued. When developing the MAPS items, we were guided by the qualitative basis that preceded the development of the Sources of Meaning and Meaning in Life Questionnaire, SoMe [11–13], by findings from the SoMe regarding highly loading items, and by the international literature on facets of meaningfulness.

Principal axis factoring revealed one-dimensional solutions for Meaningfulness and Crisis of Meaning (Study 1) and a five-factor solution for the first version of the sources of purpose scales (Study 2). Study 3, which surveyed both the MAPS and the SoMe, provided evidence for the convergent and divergent validity of the final version of the MAPS. Study 4 obtained evidence of criterion validity for the Sustainability scale, which addresses a new aspect compared to the SoMe. The data showed that sustainability as a source of purpose was associated with pro-environmental behavior, as had been expected.

Study 5 was a large-scale, population-based study, which allowed the establishment of reference values for the MAPS scales. The data also served as the basis for calculating the item and scale characteristics of the final MAPS, all of which can be described as very good. Furthermore, we examined the correlations between the meaning and purpose scales. In accordance with the theoretical model, all purpose scales correlated positively with Meaningfulness, with Personal Growth, Sustainability, and Community exhibiting large correlations, followed by Security and Faith with medium-sized correlations. This suggests that the experience of meaning is especially sustained by the pursuit of three concerns: the concern for knowing myself well and striving to further learn and mature, the concern for the well-being of those close to me, and the concern for the more-than-human world, including a perspective that extends beyond the present.

Negative correlations of the purpose scales with Crisis of Meaning were also significant, but considerably smaller. The association between Faith and Crisis of Meaning was especially minor. This suggests that a sense of connectedness with transcendence and concern for a spiritual life might also be accompanied by existential uncertainty. The so-called culture fit hypothesis offers an explanation for this finding: Studies have shown that an orientation towards religiosity and/or spirituality is primarily related with well-being if it is endorsed and valued by the majority society [72–74]. This is probably less the case in Germany, which is becoming increasingly secular [75].

Correlations between the MAPS and sociodemographic and economic variables indicated that neither the experience of meaning nor the individual sources of purpose are equally distributed across the population. It is notable, however, that people living in East Germany hardly differed from the rest of Germany in terms of meaning and purpose. Until 1990, Germany consisted of a conservative welfare state in the West and a socialist system in the East. Religious affiliation was systematically discouraged in the East, which is reflected in the lower level of Faith in our data. Various studies had also revealed a persistent satisfaction gap between East and West Germany, with individuals living in East Germany reporting less satisfaction with life than those living in the West [76]. This is not reflected in the meaning-related results. On the one hand, this could be due to the fact that life satisfaction and meaning in life refer to different aspects; on the other hand, it might be explained by the observation that various differences between East and West seem to diminish with passing time since reunification, as has been shown for, e.g., terminal decline and late-life well-being [77]. Another of our findings is new and of practical relevance: Individuals who experienced problems making ends meet financially at the end of the month reported substantially more crisis of meaning and less meaningfulness (medium effects). The sources of purpose do not seem to play a crucial role here; however, there are some indications that both the sense of connectedness with a familiar group (Community) and the connectedness with one’s self (Personal Growth) might be impaired when financial difficulties are present. The findings highlight the importance of not only focusing on the individual for understanding the construct of meaning in life, but also their integration into economic and socio-historical contexts [2].

Confirmatory factor analyses provided evidence for factorial validity in two gender-specific and two age-specific samples. Test-retest reliabilities over two time periods - four weeks and two months - were found to be excellent (Study 6). Finally, the clinical relevance of the MAPS was demonstrated by examining their predictive validity concerning general mental distress (PHQ-4). Meaningfulness and Crisis of Meaning together explained a high proportion (28%) of additional variance in general mental distress beyond sociodemographic and economic variables. Both scales also captured unique variance in the outcome variable: With higher levels of Meaningfulness, general mental distress decreased, whereas elevated scores in Crisis of Meaning were associated with higher general mental distress. In line with the literature, the sources of purpose scales explained less variance in general mental distress. All scales except for Faith were negatively related with PHQ-4 levels (small to medium zero-order correlations), but only Personal Growth emerged as a negative predictor of the core symptoms of depression and anxiety.

Although cross-sectional data cannot establish causality, this finding suggests that a commitment to a sense of connectedness to self and a concern for continued learning may be more likely to prevent anxiety and depression than the other sources of purpose appear to be. People who focus on their personal development may be less likely to be touched and affected by external conditions. This is different for the other sources of purpose. For instance, there is evidence that people who experience a stronger connectedness with the natural world suffer more from the climate crisis and report anxiety related to climate change [78]. Those who are more focused on security and safety may also be more troubled by the ongoing climate, political, economic, and health crises. People who have a strong connection to transcendence and live their lives spiritually also seem to be at risk of suffering from mental health problems [79, 80]. Finally, also being concerned for loved ones is fraught with more insecurity than focusing on one’s own advancement, as many outcomes lie outside of one’s control.

In summary, sources of purpose may provide meaning, but they do not necessarily imply health. Whatever is particularly dear to us may also cause us particular distress. In psychological practice, then, it is a matter of finding an appropriate balance between concern and worry – perhaps with attention to the “surprising upsides of worry” [81].

Since Meaningfulness shows a strong (negative) link to general mental distress, we can further postulate that the sources of purpose are associated with mental stability in those cases where they are actually experienced as providing meaning. Further studies are needed to test this hypothesis. In summary, our findings show that the MAPS are a reliable and valid measure of Meaningfulness, Crisis of Meaning, and five sources of purpose.

### Practical suggestions

Practically, it is possible to employ the entire MAPS or only individual scales. For differentiated analyses of personal sources of meaning at the individual level, more comprehensive questionnaires such as the SoMe should be used. As the MAPS and SoMe Meaningfulness and Crisis of Meaning Scales overlap strongly, they might be considered parallel measures. However, since one translation of the SoMe Meaningfulness scale showed evidence of two-dimensionality [82], the newly developed MAPS scale is preferable to the scale originating from the SoMe.

### Strengths, limitations, and constraints on generality

We have paid great attention to providing empirical backing for the development of the MAPS and including different groups of people in the validation process. Study 5, which is representative of the population in terms of gender, age, educational qualifications, and region, is of particular value in this respect. As such, it is one of the few studies that is not biased towards higher education and female gender. However, panel studies are not random samples, and they are not representative of the entire population. Citizens with little knowledge of German are not covered, which amounted to 12.1% of the working-age population in 2019 [83]. Nor are people without internet access represented by our data (2020: 4%; [84]).

Furthermore, the studies reported here refer exclusively to the validity of the German version of the MAPS. Translations and validations in English, Danish, Norwegian, and Hungarian are underway and confirm the usability of the MAPS in these contexts. However, a generalization of these results, especially to non-European cultures, is not permissible and requires further research.

## Conclusion

Based on the present studies, we were able to show that a multidimensional concept of meaning in life can be captured by highly economic scales that have proven to be reliable and valid in several respects. Hence, a short assessment of qualities of meaning (Meaningfulness and Crisis of Meaning) and five sources of purpose is made possible by the MAPS.

### Electronic supplementary material

Below is the link to the electronic supplementary material.

Supplementary Material 1

## Data Availability

The datasets used and analysed during the current study are available from the corresponding author on reasonable request.

## References

[1] Glaw X, Kable A, Hazelton M, Inder K (2017). Meaning in life and meaning of life in mental health care: an integrative literature review. Issues Ment Health Nurs.

[2] Schnell T (2021). The psychology of meaning in life.

[3] Stavrova O, Luhmann M (2016). Social connectedness as a source and consequence of meaning in life. J Posit Psychol.

[4] Leontiev DA (2007). Approaching worldview structure with ultimate meanings technique. J Humanist Psychol.

[5] Rousseau D, Billingham J (2018). A systematic framework for exploring worldviews and its generalization as a multi-purpose inquiry framework. Systems.

[6] De Vogler KL, Ebersole P (1981). Adults’ meaning in life. Psychol Rep.

[7] Wong PT, Wong PTP, Fry PS (1998). Implicit theories of meaningful life and the development of the personal meaning profile. The human quest for meaning: a handbook of psychological research and clinical applications.

[8] Delle Fave A, Brdar I, Wissing MP, Vella-Brodrick DA (2013). Sources and motives for personal meaning in adulthood. J Posit Psychol.

[9] Grouden ME, Jose PE (2015). Do sources of meaning differentially predict search for meaning, presence of meaning, and wellbeing?. Int J Wellbeing.

[10] Schnell T (2005). 73 structured exploratory interviews. [Unpublished raw data].

[11] Schnell T (2009). The sources of meaning and meaning in life questionnaire SoMe: relations to demographics and well-being. J Posit Psychol.

[12] Schnell T, Kreitler S, Urbanek T (2014). An empirical approach to existential psychology: meaning in life operationalized. Conceptions of meaning.

[13] Schnell T, Becker P (2007). Der Fragebogen zu Lebensbedeutungen und Lebenssinn LeBe.

[14] Schnell T (2004). Implizite Religiosität - Zur Psychologie des Lebenssinns.

[15] Hirsch Hadorn G, Biber-Klemm S, Grossenbacher-Mansuy W, Hoffmann-Riem H, Joye D, Pohl C, Zemp E, Hirsch Hadorn G (2008). The emergence of transdisciplinarity as a form of research. Handbook of transdisciplinary research.

[16] George LS, Park CL (2017). The multidimensional existential meaning scale: a tripartite approach to measuring meaning in life. J Posit Psychol.

[17] Martela F, Steger MF (2016). The three meanings of meaning in life: distinguishing coherence, purpose, and significance. J Posit Psychol.

[18] Delle Fave A, Soosai-Nathan L (2014). Meaning as inter-connectedness: theoretical perspectives and empirical evidence. J Psychol Afr.

[19] Schnell T, Höge T, Weber WG, Yeoman R, Bailey K, Madden A, Thompson M (2019). ‘Belonging’ and its relationship to the experience of meaningful work. The Oxford handbook of meaningful work.

[20] Stillman TF, Baumeister RF (2009). Uncertainty, belongingness, and four needs for meaning. Psychol Inq.

[21] Schnell T, Hoffmann C (2020). ME-Work: development and validation of a modular meaning in work inventory. Front Psychol.

[22] Heidegger M (1962). Being and time.

[23] Wheeler M. Martin Heidegger. In: Zalta EN, editor. The Stanford encyclopedia of philosophy. 1995. http://platostanfordedu/archives/fall2014/entries/heidegger. Accessed 02 Feb 2022.

[24] Austad A, Cheyeka AM, Danbolt LJ, Kamanga G, Mwale N, Stifoss-Hanssen H, Sørensen T, Schnell T (2023). Experiences of meaning in life in urban and rural Zambia. Arch Psychol Relig.

[25] Wissing MP (2014). Meaning and relational well-being in cross-cultural perspectives. S Afr J Psychol.

[26] Ratcliffe M, Fingerhut J, Marienberg S (2012). The phenomenology of existential feeling. Feelings of being alive.

[27] Yalom I (1980). Existential psychotherapy.

[28] Le Penne S (2017). Longing to belong: needing to be needed in a world in need. Society.

[29] Sattler J, Kearney M (2012). Belonging to the world: cosmopolitanism as a remedy against strangeness. From conflict to recognition.

[30] Schueth S, O’loughlin J (2008). Belonging to the world: cosmopolitanism in geographic contexts. Geoforum.

[31] Ratcliffe M (2009). Belonging to the world through the feeling body. Philos Psychiatry Psychol.

[32] Clifton JD (2020). Managing validity versus reliability - trade-offs in scale-building decisions. Psychol Methods.

[33] Steger MF, Frazier P, Oishi S, Kaler M (2006). The meaning in life questionnaire: assessing the presence of and search for meaning in life. J Couns Psychol.

[34] Damásio BF, Koller SH, Schnell T (2013). Sources of meaning and meaning in life questionnaire SoMe: psychometric properties and sociodemographic findings in a large brazilian sample. Acta de Investigación Psicológica.

[35] Dyrendal A, Hestad K (2021). Trust in crisis. Conspiracy mentality, lack of trust and religiosity predicted conspiracy beliefs about COVID-19 in a norwegian sample. Approaching Relig.

[36] Mavrogiorgou P, Chmielewski F, Hanning S, Juckel G, Persönlichkeit (2020). Lebensbedeutungen und Angst vor dem Tod bei affektiven Störungen. Psychotherapeut.

[37] Pedersen HF, Birkeland MH, Jensen JS, Schnell T, Hvidt NC, Sørensen T, La Cour P (2018). What brings meaning to life in a highly secular society? A study on sources of meaning among Danes. Scand J Psychol.

[38] Rahiminezhad A, Hushmand Chatroidi AM, Ejei J (2017). An investigation of the relationship between sources of meaning of life and mental health. Q Clin Psychol Stud.

[39] Schnell T, Krampe H (2020). Meaning in life and self-control buffer stress in times of COVID-19: moderating and mediating effects with regard to mental distress. Front Psychiatry.

[40] Schnell T, Krampe H (2022). Meaningfulness protects from and crisis of meaning exacerbates general mental distress longitudinally. BMC Psychiatry.

[41] Soffer Y, Wolf JJ, Ben-Ezra M (2011). Correlations between psychosocial factors and psychological trauma symptoms among rescue personnel. Prehosp Disaster Med.

[42] Sørensen T, Stifoss-Hansen H, Lien L, Pedersen HF, la Cour P, DeMarinis V, Danbolt LJ, Schnell T (2019). The sources of meaning and meaning in Life Questionnaire (SoMe) in the norwegian context: relations to mental health, quality of life and self-efficacy. Int J Psychol Relig.

[43] Valdés-Stauber J, Lemaczyk R, Kilian R (2018). Sources of meaning in family caregivers of terminally ill patients supported by a palliative nursing care team: a naturalistic three-month cohort study. Palliat Support Care.

[44] Schnell T (2010). Existential indifference: another quality of meaning in life. J Humanist Psychol.

[45] Damásio BF, Koller SH (2015). How search for meaning interacts with complex categories of meaning in life and subjective well-being?. Span J Psychol.

[46] Jantzen W. Am Anfang war der Sinn. Zur Naturgeschichte, Psychologie und Philosophie von Tätigkeit, Sinn und Dialog. BdWi-Verlag; 1994.

[47] McDonald MJ, Wong PT, Gingras DT, Wong PT (2012). Meaning-in-life measures and development of a brief version of the Personal meaning Profile. The human quest for meaning.

[48] Lavigne KM, Hofman S, Ring AJ, Ryder AG, Woodward TS (2013). The personality of meaning in life: associations between dimensions of life meaning and the big five. J Posit Psychol.

[49] Schnell T, Becker P (2006). Personality and meaning in life. Pers Individ Differ.

[50] Faul F, Erdfelder E, Lang AG, Buchner A (2007). G* power 3: a flexible statistical power analysis program for the social, behavioral, and biomedical sciences. Behav ResMethods.

[51] Pett MA, Lackey NR, Sullivan JJ (2003). Making sense of factor analysis: the use of factor analysis for instrument development in health care research.

[52] Haynes SN, Richard D, Kubany ES (1995). Content validity in psychological assessment: a functional approach to concepts and methods. Psychol Assess.

[53] Schnell T, Krampe H (2015). Alcohol, drugs, and smoking among future psychologists: differential contributions of big five personality traits, sources of meaning, and self-efficacy. Clin Health Promot.

[54] Spitzenstätter D, Schnell T (2022). Effects of mortality awareness on attitudes toward dying and death and meaning in life-a randomized controlled trial. Death Stud.

[55] Vötter B, Schnell T (2019). Cross-lagged analyses between life meaning, self-compassion, and subjective well-being among gifted adults. Mindfulness.

[56] EPA. Learn about sustainability. https://wwwepa.gov/sustainability/learn-about-sustainability#what (2021). Accessed 5 December 2022.

[57] SurveyMonkey. (2023). Repräsentative Stichprobe berechnen: Formeln, Beispiele und Tipps. [How to estimate a representative sample: formulas, examples and tips] https://www.surveymonkey.de/mp/repraesentative-stichprobe-berechnen-formeln-beispiele-und-tipps/ Accessed 01 August 2023.

[58] Leiner DJ (2019). Too fast, too straight, too weird: non-reactive indicators for meaningless data in internet surveys. Surv Res Methods.

[59] Kuivalainen S, Michalos AC (2014). Subjective poverty. Encyclopedia of quality of life and well-being research.

[60] Hayes AF, Coutts JJ (2020). Use omega rather than Cronbach’s alpha for estimating reliability. But… Commun Methods Meas.

[61] Rammstedt B, Beierlein C (2014). Can’t we make it any shorter? The limits of personality assessment and ways to overcome them. J Individ Differ.

[62] George D, Mallery P. IBM SPSS statistics 26 step by step: a simple guide and reference. Routledge; 2019.

[63] Sullins DP (2006). Gender and deconstructing universality, constructing complexity. Am J Sociol.

[64] Schnell T (2015). Dimensions of Secularity (DoS): an open inventory to measure facets of secular identities. Int J Psychol Relig.

[65] Twenge JM (2009). Status and gender: the paradox of progress in an age of narcissism. Sex Roles.

[66] Kyriazos T (2018). Applied psychometrics: sample size and sample power considerations in factor analysis (EFA, CFA) and SEM in general. Psychology.

[67] Schermelleh-Engel K, Moosbrugger H, Müller H (2003). Evaluating the fit of structural equation models: tests of significance and descriptive goodness-of-fit measures. Methods Psychol Res Online.

[68] Cicchetti DV (1994). Guidelines, criteria, and rules of thumb for evaluating normed and standardized assessment instruments in psychology. Psychol Assess.

[69] Carreno DF, Eisenbeck N, Cangas AJ, García-Montes JM, Del Vas LG, María AT (2020). Spanish adaptation of the Personal meaning Profile-Brief: meaning in life, psychological well-being, and distress. Int J Clin Hlth Psyc.

[70] Kroenke K, Spitzer RL, Williams JB, Löwe B (2009). An ultra-brief screening scale for anxiety and depression: the PHQ–4. Psychosomatics.

[71] Keenan WJ (2020). Learning to survive: wicked problem education for the anthropocene age. Glob Res J Educ.

[72] Diener E, Tay L, Myers DG (2011). The religion paradox: if religion makes people happy, why are so many dropping out?. J Pers Soc Psychol.

[73] Gebauer JE, Sedikides C, Schönbrodt FD, Bleidorn W, Rentfrow PJ, Potter J, Gosling SD (2017). The religiosity as social value hypothesis: a multi-method replication and extension across 65 countries and three levels of spatial aggregation. J Pers Soc Psychol.

[74] Lun VMC, Bond MH (2013). Examining the relation of religion and spirituality to subjective well-being across national cultures. Psychol Relig.

[75] Fowid. Religionszugehörigkeiten. https://fowid.de/meldung/religionszugehoerigkeiten-2020 (2021). Accessed 1 August 2023.

[76] Biermann P, Welsch H (2021). An anatomy of east german unhappiness: the role of circumstances and mentality, 1990–2018. J Econ Behav Organ.

[77] Vogel N, Gerstorf D, Ram N, Goebel J, Wagner GG (2017). Terminal decline in well-being differs between residents in East Germany and West Germany. Int J Behav Dev.

[78] Coffey Y, Bhullar N, Durkin J, Islam MS, Usher K (2021). Understanding eco-anxiety: a systematic scoping review of current literature and identified knowledge gaps. J Clim Chang Health.

[79] King M, Marston L, McManus S, Brugha T, Meltzer H, Bebbington P (2013). Religion, spirituality and mental health: results from a national study of English households. BJPsych.

[80] Schnell T (2012). Spirituality with and without religion—Differential relationships with personality. Arch Psychol Relig.

[81] Sweeny K, Dooley MD (2017). The surprising upsides of worry. Soc Personal Psychol Compass.

[82] Damásio BF, Hauck-Filho N, Koller SH (2016). Measuring meaning in life: an empirical comparison of two well-known measures. J Happiness Stud.

[83] Grotlüschen A, Buddeberg K, editors. LEO 2018: Leben mit geringer Literalität. wbv; 2020.

[84] Eurostat. : Haushalte – Internet-Zugangsdichte [Online data code: ISOC_CI_IN_H] https://ec.europa.eu/eurostat/databrowser/view/ISOC_CI_IN_H/default/table?lang=de (2022). Accessed 5 April 2022.

