# Supplementary material for: The Meaning and Purpose Scales (MAPS): development and multi-study validation of short measures of meaningfulness, crisis of meaning, and sources of purpose
Source: BMC Psychol. 2023 Oct 3;11:304. doi: 10.1186/s40359-023-01319-8 (PMC10548553; doi:10.1186/s40359-023-01319-8)
Supplement: Supplementary file 1 — Supplementary Material 1 [file 40359_2023_1319_MOESM1_ESM.docx]

# **Appendix**

Table A1 presents the original German version, table A2 presents an English translation of the MAPS.

**Table A1**

*MAPS Items – Deutsche Version (German Version)*

| **German version** (Deutsche Version) | |
| --- | --- |
| **Sinnerfüllung** - die grundlegende Erfahrung, dass das Leben lebenswert ist, basierend auf der Bewertung des eigenen Lebens als orientiert, kohärent, zugehörig und bedeutsam | |
| 1 | Mein Leben ist sinnvoll. |
| 2 | Ich habe meinen Weg gefunden. |
| 3 | Mein Leben erscheint mir stimmig. |
| 4 | Ich fühle mich dieser Welt verbunden. |
| 5 | Mein Dasein bereichert das Leben von anderen. |
| **Sinnkrise** - die Bewertung des eigenen Lebens als frustrierend leer, ohne Sinn und Bedeutung | |
| 1 | Ich leide darunter, dass ich keinen Sinn in meinem Leben sehen kann. |
| 2 | Ich vermisse einen Sinn in meinem Leben. |
| 3 | Mein Leben erscheint mir leer. |
| **Nachhaltigkeit** - Verbundenheit mit allem Lebendigen und Sorge für eine lebenswerte Zukunft | |
| 1 | Ich richte mein Handeln danach aus, den nächsten Generationen eine lebenswerte Welt zu hinterlassen. |
| 2 | Ich achte sehr darauf, im Einklang mit der Natur zu leben. |
| 3 | Ich erlebe eine starke Verbundenheit mit allen Lebewesen auf der Erde. |
| **Glaube** - Transzendenzverbundenheit und Sorge für ein spirituelles Leben | |
| 1 | Ich fühle mich gestärkt durch den Glauben an Gott/eine höhere Macht. |
| 2 | Es ist wichtig für mich, mit Gott/einer höheren Macht in Kontakt zu treten. |
| 3 | Mein alltägliches Handeln wird durch den Glauben an Gott/eine höhere Macht bestimmt. |
| **Sicherheit** - Verbundenheit mit gemeinsamen Normen und Sorge für ein sicheres Leben | |
| 1 | Ich halte mich immer an Regeln und Vorschriften. |
| 2 | Bei Entscheidungen wähle ich immer den sicheren Weg. |
| 3 | Ich handle immer vernünftig und mit Bedacht. |
| **Gemeinschaftssinn** - Verbundenheit mit einer vertrauten Gruppe und Sorge für das Miteinander | |
| 1 | Ich finde es wichtig, mich um das Wohlgefühl der Menschen um mich herum zu kümmern. |
| 2 | Ich verbringe gern Zeit in Gemeinschaft mit anderen Menschen. |
| 3 | Die Nähe zu anderen Menschen ist die Grundlage eines guten Lebens. |
| **Persönliches Wachstum** - Selbstverbundenheit und Sorge für anhaltendes Lernen | |
| 1 | Ich arbeite stets daran, persönlich zu wachsen und mich weiterzuentwickeln. |
| 2 | Ich setze mir Ziele, durch die ich weiter lernen kann und muss. |
| 3 | Ich setze mich gründlich mit meinen Interessen, Stärken und Schwächen auseinander. |

*Anmerkung*. Antwortformat = sechsstufige Likert-Skala (0 = stimme gar nicht zu bis 5 = stimme vollkommen zu).

**Table A2**

*MAPS Items - English Version (Ad Hoc Translation)*

| **English version** | |
| --- | --- |
| **Meaningfulness** - the basic sense that life is worth living, based on the evaluation of one’s life as directed, coherent, belonging, and significant | |
| 1 | My life is meaningful. |
| 2 | I have found my way. |
| 3 | My life makes sense to me. |
| 4 | I feel connected to this world. |
| 5 | My existence enriches the life of others. |
| **Crisis of Meaning** - a judgement on one’s life as frustratingly empty, pointless, and lacking meaning | |
| 1 | I am missing meaning in my life. |
| 2 | I suffer because I can't see any meaning in my life. |
| 3 | My life seems empty to me. |
| **Sustainability** - a sense of connectedness with all forms of life and concern for a future worth living | |
| 1 | I base my actions on leaving a world worth living in for the next generations. |
| 2 | I take great care to live in harmony with nature. |
| 3 | I experience a strong connection with all living beings on earth. |
| **Faith** - a sense of connectedness with transcendence and concern for a spiritual life | |
| 1 | I find strength through faith in God/a higher power. |
| 2 | It is important for me to connect with God/a higher power. |
| 3 | My everyday actions are guided by faith in God/a higher power. |
| **Security** - a sense of connectedness with shared norms and concern for a secure life | |
| 1 | I always follow rules and regulations. |
| 2 | When making decisions, I always go for the safe option. |
| 3 | My actions are sensible and prudent at all times. |
| **Community** - a sense of connectedness with a familiar group and concern for each other | |
| 1 | I find it important to care for the well-being of the people around me. |
| 2 | I enjoy spending time in community with other people. |
| 3 | Being close to other people is the foundation of a good life. |
| **Personal Growth** - a sense of connectedness with one’s self and concern for continuous learning | |
| 1 | I am constantly working to grow and develop personally. |
| 2 | I set myself goals that encourage me to continue learning. |
| 3 | I thoroughly explore my interests, strengths, and weaknesses. |

*Note*. Response format = six-point Likert-scale (0 = /totally disagree to 5 = totally agree).

Table A3 shows age-specific reference scores. Because correlations between the MAPS and gender were small to non-existent (see Tab. 6), no gender-specific reference scores are given.

**Table A3**

*MAPS Means and Standard Deviations and Frequencies of Types of Meaning for Four Age Groups*

|  | Total sample^a^ | 18-29^b^ | 30-46^c^ | 47-64^d^ | 65-89^e^ |
| --- | --- | --- | --- | --- | --- |
| **Scales** | Mean (SD) | | | | |
| Meaningfulness^b^ | 3.37 (0.91) | 3.09 (0.78) | 3.20 (0.94) | 3.42 (0.90) | 3.69 (0.87) |
| Crisis of Meaning^b^ | 1.19 (1.30) | 1.70 (1.33) | 1.49 (1.35) | 1.07 (1.30) | 0.69 (0.92) |
| Sustainability^b^ | 3.08 (1.11) | 2.67 (1.13) | 2.77 (1.09) | 3.21 (1.07) | 3.52 (0.98) |
| Faith^b^ | 1.47 (1.68) | 1.22 (1.58) | 1.34 (1.55) | 1.50 (1.72) | 1.74 (1.80) |
| Security^b^ | 3.30 (0.90) | 3.29 (0.83) | 3.17 (0.92) | 3.30 (0.91) | 3.51 (0.88) |
| Community^b^ | 3.44 (1.02) | 3.52 (0.83) | 3.35 (0.98) | 3.38 (1.06) | 3.65 (1.04) |
| Personal Growth^b^ | 3.43 (1.00) | 3.48 (0.97) | 3.41 (1.03) | 3.43 (0.97) | 3.43 (1.03) |
| **Meaning Types** | % | | | | |
| “Meaningfulness” | 68 | 57 | 61 | 69 | 81 |
| “Crisis of Meaning” | 14 | 23 | 20 | 13 | 4 |
| “Existential Indifference” | 18 | 20 | 19 | 18 | 15 |

*Note.* ^a^ *N* = 974. ^b^ *N* = 115. ^c^ *N* = 284. ^d^ *N* = 387. ^e^ *N* = 188.

Table A4 shows means and standard deviations for responses to the individual MAPS questions based on the entire sample.

**Table A4**

*MAPS Items’ Means and Standard Deviations*

|  | Mean | *SD* |
| --- | --- | --- |
| **Meaningfulness** |  |  |
| My life is meaningful. | 3.53 | 1.29 |
| I have found my way. | 3.36 | 1.28 |
| My life makes sense to me. | 3.24 | 1.22 |
| I feel connected to this world. | 3.28 | 1.27 |
| My existence enriches the life of others. | 3.12 | 1.24 |
| **Crisis of Meaning** |  |  |
| I am missing meaning in my life. | 1.09 | 1.39 |
| I suffer because I can’t see any meaning in my life. | 1.37 | 1.50 |
| My life seems empty to me. | 1.12 | 1.41 |
| **Sustainability** |  |  |
| I base my actions on leaving a world worth living in for the next generations. | 3.21 | 1.28 |
| I take great care to live in harmony with nature. | 3.22 | 1.28 |
| I experience a strong connection with all living beings on earth. | 2.82 | 1.40 |
| **Faith** |  |  |
| I find strength through faith in God/a higher power. | 1.67 | 1.80 |
| It is important for me to connect with God/a higher power. | 1.47 | 1.79 |
| My everyday actions are guided by faith in God/a higher power. | 1.27 | 1.65 |
| **Security** |  |  |
| I always follow rules and regulations. | 3.40 | 1.10 |
| When making decisions, I always go for the safe option. | 3.26 | 1.10 |
| My actions are sensible and prudent at all times. | 3.24 | 1.14 |
| **Community** |  |  |
| I find it important to care for the well-being of the people around me. | 3.66 | 1.13 |
| I enjoy spending time in community with other people. | 3.30 | 1.34 |
| Being close to other people is the foundation of a good life. | 3.35 | 1.22 |
| **Personal Growth** |  |  |
| I am constantly working to grow and develop personally. | 3.58 | 1.18 |
| I set myself goals that encourage me to continue learning. | 3.32 | 1.23 |
| I thoroughly explore my interests, strengths, and weaknesses. | 3.39 | 1.13 |

*Note.* *N* = 974.
